# A comparative analysis of DeepSeek R1, DeepSeek-R1-Lite, OpenAi o1 Pro, and Grok 3 performance on ophthalmology board-style questions

**DOI:** 10.1038/s41598-025-08601-2

**Published:** 2025-07-02

**Authors:** Ryan Shean, Tathya Shah, Aditya Pandiarajan, Alan Tang, Kyle Bolo, Van Nguyen, Benjamin Xu

**Affiliations:** 1 Keck School of Medicine, University of Southern California, 1975 Zonal Avenue, Los Angeles, CA USA; 2 Information Sciences Institute, University of Southern California, 4676 Admiralty Way #1001, Marina Del Rey, CA USA; 3 Keck School of Medicine, Roski Eye Institute, University of Southern California, 1450 San Pablo Street, Los Angeles, CA USA; 4 Department of Ophthalmology, Keck School of Medicine, University of Southern California, 1450 San Pablo Street, 4th Floor, Suite 4700, Los Angeles, CA 90033 USA

**Keywords:** Artificial intelligence, Ophthalmology, Medical education, Large Language models, Health care, Medical research

## Abstract

The ability of large language models (LLMs) to accurately answer medical board-style questions reflects their potential to benefit medical education and real-time clinical decision-making. With the recent advance to reasoning models, the latest LLMs excel at addressing complex problems in benchmark math and science tests. This study assessed the performance of first-generation reasoning models—DeepSeek’s R1 and R1-Lite, OpenAI’s o1 Pro, and Grok 3—on 493 ophthalmology questions sourced from the *StatPearls* and *EyeQuiz* question banks. o1 Pro achieved the highest overall accuracy (83.4%), significantly outperforming DeepSeek R1 (72.5%), DeepSeek-R1-Lite (76.5%), and Grok 3 (69.2%) (*p* < 0.001 for all pairwise comparisons). o1 Pro also demonstrated superior performance in questions from eight of nine ophthalmologic subfields, questions of second and third order cognitive complexity, and on image-based questions. DeepSeek-R1-Lite performed the second best, despite relatively small memory requirements, while Grok 3 performed inferiorly overall. These findings demonstrate that the strong performance of the first-generation reasoning models extends beyond benchmark tests to high-complexity ophthalmology questions. While these findings suggest a potential role for reasoning models in medical education and clinical practice, further research is needed to understand their performance with real-world data, their integration into educational and clinical settings, and human-AI interactions.

**Supplementary Information:**

The online version contains supplementary material available at 10.1038/s41598-025-08601-2.

## Introduction

Generative artificial intelligence (AI) is a category of AI that learns patterns from large datasets and uses these learned patterns to produce new content, including text, images, music, or videos. Large language models (LLMs) are a subset of generative AI that form the foundation of advanced chatbot tools such as OpenAI’s ChatGPT. LLMs have an emerging role in medicine to assist with medical education and clinical decision-making, offering potential assistance in areas such as board exam preparation and differential diagnosis generation^1,2^. Assessing the accuracy and reliability of LLMs in complex scientific reasoning is crucial for their safe and effective integration into both medical education and clinical practice^3–7^. In the United States, the field of ophthalmology relies on multiple-choice standardized exams administered by the American Board of Ophthalmology to assure the public that practitioners have the medical knowledge, clinical judgment, and professionalism required to provide high-quality patient care^8^.

Recent research has demonstrated that newer generations of LLMs perform better than earlier models on medical board-style questions^9–14^. Models designed for complex reasoning, including OpenAI o1 Pro and Grok 3, have traditionally been associated with significant computational resources and costs^15^. However, a novel model from DeepSeek achieved high benchmark performance despite substantially lower training costs and computational demands^16^. Two reasoning models are now available for use: DeepSeek R1, the full model capable of the most complex reasoning, and DeepSeek-R1-Lite, a smaller, more computationally efficient model (1.5 billion to 70 billion parameters compared with 671 billion parameters in the full model) that can be downloaded and run on personal computers.

This study evaluated the performance of four reasoning models—DeepSeek R1, DeepSeek-R1-Lite (15 billion parameters), o1 Pro, and Grok 3—on ophthalmology board-style questions. We hypothesized that DeepSeek R1 would perform comparably to o1 Pro and Grok 3, while outperforming DeepSeek-R1-Lite, due to its more robust architecture and known benchmark performance^16^. Clarifying the performance of these models could provide insight into the feasibility of using reasoning models, including cost-efficient and lower-memory models, in medical education and clinical decision-making^14,17^.

## Results

**Fig. 1:**
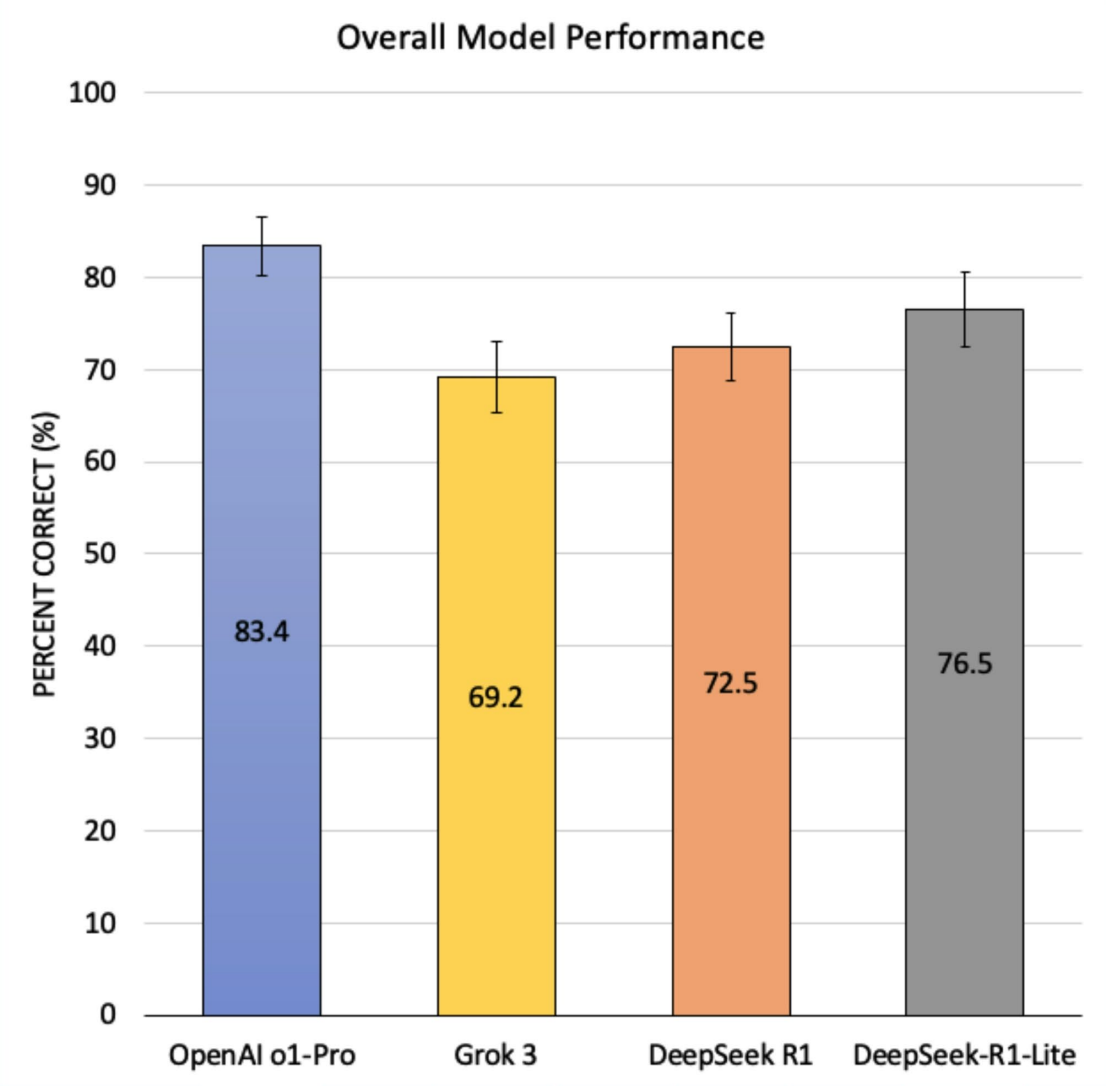
Overall performance of OpenAI o1, Grok 3, DeepSeek R1, and DeepSeek-R1-Lite on *StatPearls* and *EyeQuiz* Questions, with 95% confidence intervals included. Each color represents a distinct LLM, as noted at the bottom of the figure.

A total of 493 ophthalmology questions were analyzed—250 from *StatPearls* and 243 from *EyeQuiz*. OpenAI o1 Pro demonstrated the highest overall accuracy, correctly answering 411/493 questions (83.4%, 95% confidence interval (CI): 80.3–86.8%). DeepSeek-R1-Lite ranked second, correctly answering 377/493 (76.5%, CI: 73.0–80.4%), followed by DeepSeek R1 at 357/493 (72.5%, CI: 68.7–76.5%) and Grok 3 at 341/493 (69.2%, CI: 65.3–73.4%) (Fig. 1). Pairwise comparison demonstrated that Open AI o1 Pro significantly outperformed DeepSeek R1 (*p* < 0.001), DeepSeek-R1-Lite (*p* < 0.001), and Grok 3 (*p* < 0.001). DeepSeek-R1-Lite performed significantly better than DeepSeek R1 (*p* = 0.02) and Grok 3 (*p* = 0.001). There was no significant difference in overall performance between DeepSeek R1 and Grok 3 (*p* = 0.12).

**Fig. 2:**
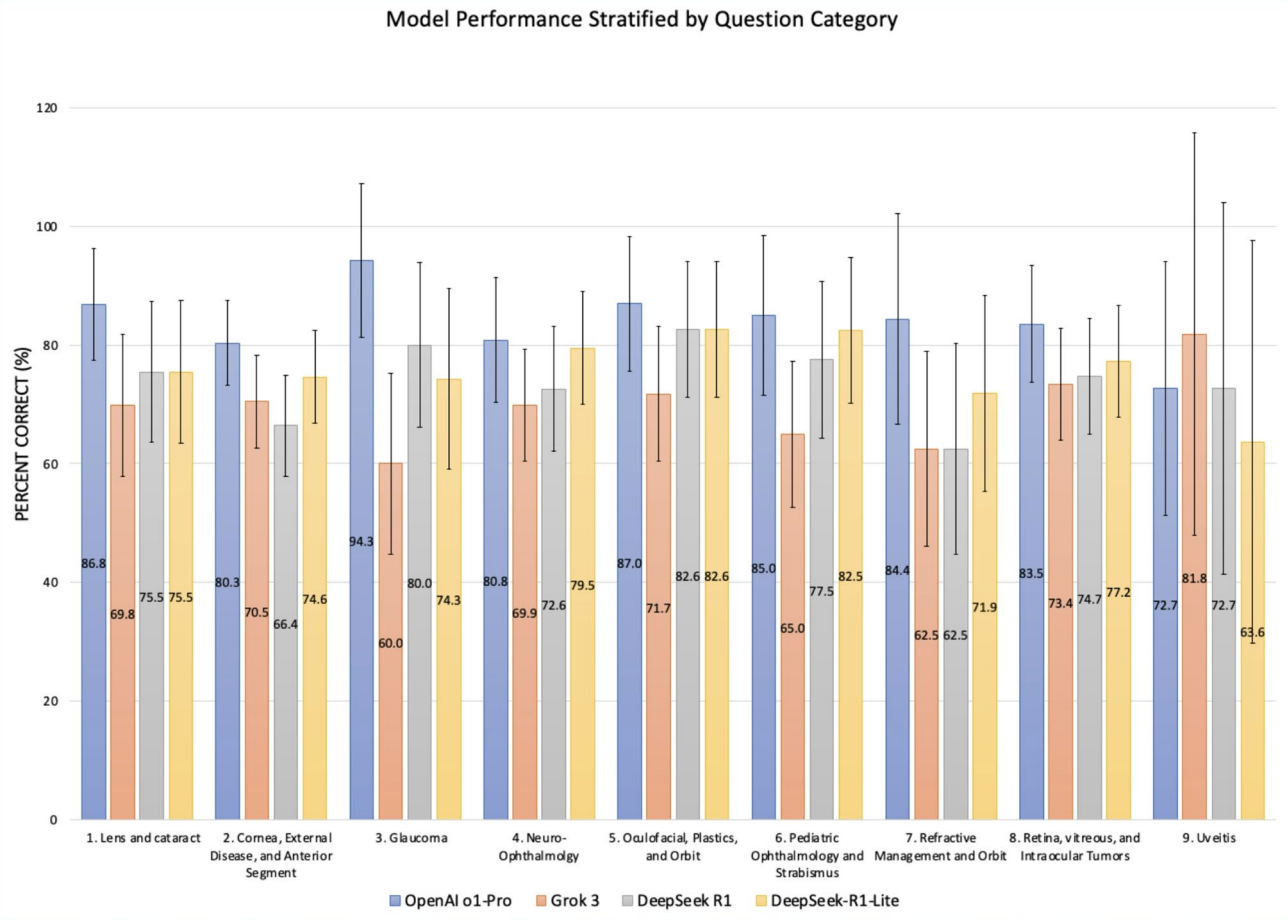
Performance of OpenAI o1, Grok 3, DeepSeek R1, and DeepSeek-R1-Lite on *StatPearls* and *EyeQuiz* Questions, Stratified by Buckwalter Taxonomic Schema. First-order: recognition and recall, second-order: comprehension and interpretation, third-order: application of knowledge. 95% confidence intervals are included. Each color represents a distinct LLM, as noted at the bottom of the figure.

When performance was stratified by ophthalmology subfields (Fig. 2), OpenAI o1 Pro demonstrated superior accuracy in eight of the nine subcategories. The only exception was the Uveitis category (*N* = 11), where OpenAI o1 Pro had the second-highest performance. Notably, in the category containing the largest number of questions—Cornea, External Disease, and Anterior Segment (*N* = 122)—OpenAI o1 Pro correctly answered 96/122 (80.3%, CI: 73.1–87.4%), compared to DeepSeek-R1-Lite at 91/122 (74.6%, CI: 66.8–82.5%), Grok 3 at 86/122 (70.5%, CI: 62.7–78.3%), and DeepSeek R1 at 81/122 (66.4%, CI: 57.9–74.9%).

**Fig. 3:**
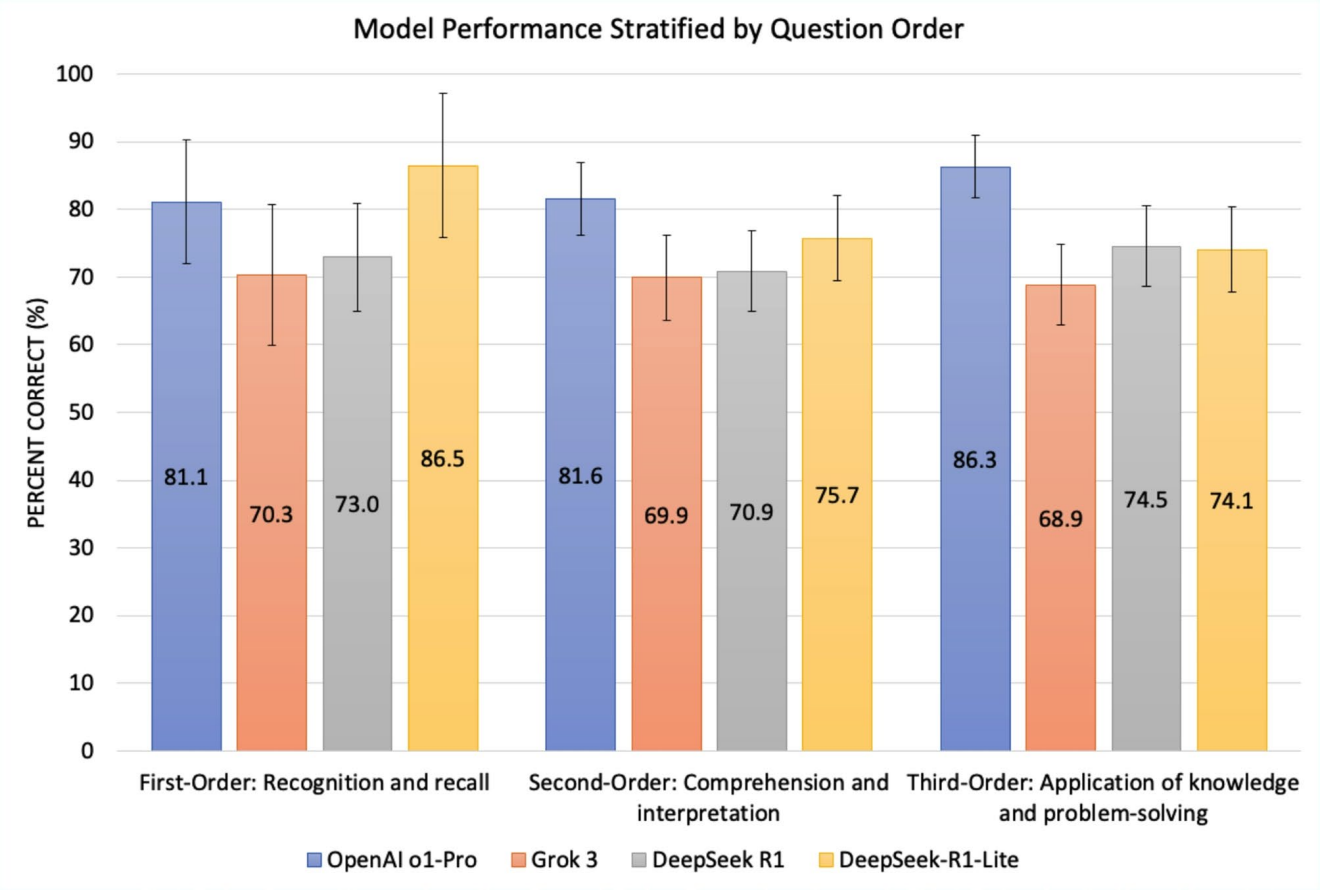
Performance of OpenAI o1, Grok 3, DeepSeek R1, and DeepSeek-R1-Lite on *StatPearls* and *EyeQuiz* questions stratified into nine distinct ophthalmology subfields: (1) Lens and Cataract, (2) Cornea, External Disease, and Anterior Segment, (3) Glaucoma, (4) Neuro-Ophthalmology, (5) Oculofacial, Plastics, and Orbit, (6) Pediatric Ophthalmology and Strabismus, (7) Refractive Management and Surgery, (8) Retina, Vitreous, and Intraocular Tumors, and (9) Uveitis. Each color represents a distinct LLM, as noted at the bottom of the figure.

When analyzing performance by Buckwalter’s taxonomy of cognitive complexity (Fig. 3), DeepSeek-R1-Lite achieved the highest accuracy for first-order (recognition and recall) questions, correctly answering 64/74 (86.5%, CI: 79.7–93.3%). OpenAI o1 Pro followed with 60/74 (81.1%, CI: 73.6–88.6%, *p* = 0.12). OpenAI o1 Pro exhibited the best performance for second-order (comprehension and interpretation) and third-order (application of knowledge and problem-solving) questions. OpenAI-o1 Pro’s strongest performance was observed in third-order questions, with 183/212 correct responses (86.3%, CI: 81.5–91.1%), representing an 11.8% advantage over DeepSeek R1 (74.1%, CI: 68.2–80.0%, *p* < 0.001). For the second-order questions, OpenAI o1 Pro answered correctly 168/206 (81.6%, CI: 76.3–86.9%), followed by DeepSeek-R1-Lite 156/206 (75.7%, CI: 70.2–81.2%, *p* = 0.05), DeepSeek R1 with 146 (70.9%, CI: 64.8–77.0%, *p* < 0.001), and Grok 3 at 144/206 (69.9%, CI: 63.8–76.0%, *p* < 0.001).

Comparing performance on image-based versus non-image-based questions, OpenAI o1 Pro maintained superior accuracy, correctly answering 61/74 image-based questions (82.4%, CI: 73.5–91.3%) and 351/419 non-image questions (83.8%, CI: 80.3–87.3%) (Supplementary Figure S1). While all models performed better on non-image questions than image questions, OpenAI o1 Pro exhibited the smallest discrepancy (1.4%) between accuracy for image and non-image questions. In contrast, Grok 3 displayed the largest performance gap of 11.6%, followed by DeepSeek-R1-Lite with a 6.0% gap, and DeepSeek R1 with a 4.4% gap.

Each LLM model was also evaluated according to performance between the *EyeQuiz* and *StatPearls* question banks (Supplementary Figure S2). Grok 3 and DeepSeek R1 demonstrated very similar accuracy rates across both question banks. OpenAI o1 Pro answered a greater percentage of *StatPearls* than *EyeQuiz* questions correctly (86.7% compared to 80.4%), though this difference was not statistically significant (*p* = 0.06). DeepSeek-R1-Lite answered a greater percentage of *EyeQuiz* than *StatPearls* questions correctly (80.0% compared to 73.4%) on *StatPearls* questions, though this difference was also not statistically significant (*p* = 0.76).

## Discussion

Our findings demonstrate that OpenAI o1 Pro outperformed DeepSeek R1, DeepSeek-R1-Lite, and Grok 3 in overall accuracy, as well as across nearly all ophthalmology subfields and levels of cognitive complexity. Notably, o1 Pro showed superior performance on second- and third-order questions, indicating stronger complex reasoning capabilities. While o1 Pro was the top performer overall (83.4%), DeepSeek-R1-Lite performed respectably, achieving the second-highest accuracy (76.5%) and excelling specifically in first-order recall-based questions.

OpenAI o1 Pro, DeepSeek R1, and DeepSeek-R1-Lite each achieved higher accuracy than previously reported LLM performance on ophthalmology board-style question banks (46.7–71%)^18,19^. One possible explanation for o1 Pro’s high performance may be its broader training data, stronger reasoning capabilities, or greater exposure to ophthalmology-specific content. However, its performance comes at a cost—200 United States Dollars (USD) per month for full access—which limits its accessibility to the general public. In contrast, DeepSeek-R1-Lite, despite being a smaller and more computationally-efficient model with 15 billion parameters, performed competitively. DeepSeek-R1-Lite’s balance of strong performance, low memory requirements, and affordability suggests that it may be possible to achieve LLM-based educational support without relying exclusively on the most expensive, high-resource model. As computational efficiency improves and development costs decrease, LLMs have the potential to enhance medical training by offering scalable, cost-effective, and increasingly accurate educational support^20^. For example, a medical trainee struggling with retina questions could use a highly-accurate, low-cost model for identification of knowledge gaps.

An unanticipated result was that DeepSeek-R1-Lite outperformed the full DeepSeek R1 model, despite R1’s larger architecture and enhanced parameters. This contradicted our initial hypothesis and may be explained by differences in architectural optimization between the models or R1 “overthinking” questions leading to reduced accuracy. R1-Lite’s strength in first-order questions may suggest more targeted exposure to factual ophthalmology content during training. Importantly, this performance shows that smaller models capable of running on personal devices can achieve high accuracy, challenging the assumption that extensive computational resources are essential for strong LLM performance. Furthermore, the ability to use these models offline expands their utility in settings with limited or restricted internet access, such as remote medicine, military medicine, disaster response, and even space medicine, where reliable offline AI assistance could assist decision-making.

Breaking down performance by subspecialty, OpenAI o1 Pro performed the best in eight out of nine ophthalmic categories, followed by DeepSeek-R1-Lite. While Grok 3 performed the best on Uveitis questions, this was the smallest subfield (*n* = 11) and most susceptible to variance, which may have affected the results. Overall, performance among models was relatively consistent across most subspecialties. Minor variations between models could be due to differences in training data or reasoning processes^21^. Notably, the Uveitis section contained a relatively small sample size, limiting statistical power and interpretability. Therefore, findings in this subfield should be interpreted cautiously.

Breaking down performance by cognitive complexity, OpenAI o1 Pro performed best in second- and third-order reasoning tasks, which require increased comprehension, interpretation, and problem-solving. DeepSeek-R1-Lite outperformed all models in first-order recall-based questions. This suggests that DeepSeek-R1-Lite may be optimized for fact retrieval, while OpenAI o1 Pro’s superior contextual understanding enables stronger performance in higher-order reasoning tasks. Open AI o1 Pro also scored a higher absolute percentage on third-order questions than first- and second-order questions. In contrast, Grok 3 and DeepSeek R1 performed comparably across all three cognitive complexity levels. The performance differences observed here might highlight the potential benefits DeepSeek R1’s mixture of experts (MoE) approach, where different subsystems or “experts” within the model specialize in distinct tasks or domains. This approach could lead to a more efficient use of resources and could ultimately enhance the flexibility and scalability of these models.

Across all models, image-based questions were more challenging than non-image-based questions. OpenAI o1 Pro exhibited the smallest performance gap between its image-based and non-image-based accuracy, with only a 1.4% difference. In contrast, Grok 3 had the largest discrepancy, with an 11.6% drop in accuracy for image-based questions. OpenAI o1 Pro also performed over 10% higher than all other models on image-based performance overall. This suggests that OpenAI o1 Pro may be better at analyzing visual inputs and integrating this information into its reasoning, contributing to its relative strength across advanced cognitive complexities and subspecialties. OpenAI o1 Pro employs reinforcement learning techniques and chain-of-thought prompting to facilitate multi-step reasoning processes. This architecture enables o1 Pro to explore various strategies dynamically, correct mistakes, and provide more consistent and sophisticated responses, explaining its better performance on both image-based and non-image-based questions. While Grok 3 can summarize and analyze multimodal content, its lower performance on image-based medical questions could be explained by an inadequate use of medical-specific image data in its training datasets. This large performance drop may reflect Grok 3’s limitations in effectively processing visual information, potentially due to limited exposure to medical images during training.

A key strength of this study was the use of two independent question banks, *EyeQuiz* and *StatPearls*, to validate model performance. Most other studies analyzing LLM performance on board-style questions uses one style of question from a single source^9–12,19^. Using multiple question banks provided a broader range of question styles and minimized bias from one single source of questions. By employing board-style questions, this study assesses the competency of these AI models in the same format that residents use to prepare for exams, providing insight into their potential role in assisting medical education. Grok 3 and DeepSeek R1 performed similarly across both question banks, while DeepSeek-R1-Lite performed slightly better on *EyeQuiz* and Open AI o1 Pro performed slightly better on *StatPearls*. The consistency in performance among all models across both question banks demonstrates that these LLMs were able to perform at a high accuracy across a variety of questions, and that LLM performance did not vary greatly depending on question style. Future studies should consider evaluating models on proprietary or institution-specific question sets to confirm generalizability as current benchmarks may overlap with publicly available data used to train the tested LLMs.

Our study has some limitations. First, each question was entered into each model only once. Given the probabilistic nature of LLMs, their responses can vary slightly if the same question is presented multiple times, which could affect the reproducibility of our findings. However, the large number of questions included in this study makes it unlikely that minor response variations would significantly alter overall performance trends. Future studies should consider multiple independent runs per model to assess variance. Second, our study may be limited in its applicability to clinical practice as the AI models were tested in a controlled setting that does not fully reflect real-world clinical complexities, such as synthesizing incomplete patient histories or integrating imaging and laboratory data^22,23^. Third, LLMs may produce hallucinations or factually incorrect outputs, posing a risk in high-stakes clinical settings. Ethical concerns around bias, data provenance, and informed consent must also be considered. Fourth, our LLM prompt was relatively simple, and additional prompt engineering may improve model performance. Future work could explore structured prompting techniques such as chain-of-thought, multi-step reasoning scaffolds, or clinical context reinforcement to enhance model accuracy. Finally, the ‘black box’ nature of LLMs makes it difficult to interpret how conclusions are reached, which could be a barrier to gaining clinician trust and adoption.

In conclusion, OpenAI o1 Pro outperformed DeepSeek R1, DeepSeek-R1-Lite, and Grok 3 in answering ophthalmology board-style questions spanning multiple subfields and varying levels of cognitive complexity. DeepSeek-R1-Lite and DeepSeek R1 both performed better than Grok 3, and DeepSeek-R1-Lite surprisingly outperformed DeepSeek R1 despite having significantly lower memory requirements. These findings suggest that reasoning models have the capability of handling complex ophthalmologic scenarios, and lighter models may become viable tools for use in the clinic and in medical education from the perspective of computational requirements and cost. As AI-assisted learning becomes more integrated into medical training, generative AI models could enhance study efficiency and exam preparedness^24,25^. Future research should focus on validating the strong performance of reasoning models using real-world clinical data, assessing the impact of AI-based study aids on learner outcomes, and developing strategies to integrate reasoning models into medical education and patient care.

## Methods

### Study design and AI model implementation

This research utilized four advanced reasoning artificial intelligence (AI) models—OpenAI o1 Pro, Grok 3, DeepSeek R1, and DeepSeek-R1-Lite, the latter being an offline version of DeepSeek—to assess performance on ophthalmology board-style questions (Supplementary Figure S3). The models were all accessed in March of 2025. DeepSeek-R1-Lite was downloaded and run on a personal computer (Apple M2 chip with 8-core CPU with 4 performance cores and 4 efficiency cores, 8-core GPU, 16-core Neural Engine, 100GB/s memory bandwidth), and the DeepSeek-R1-Lite version downloaded was the most recently available Lite version as of April 3, 2025 with 15 billion parameters and a size of 32 gigabytes. DeepSeek V3 was excluded from the analysis as the focus of this study was to compare the performance of LLMs designed for advanced reasoning capabilities.

Each model independently evaluated the full set of 493 questions. Questions were administered in batches of ten, except for image-based questions, which were run individually. A new chat session was initiated for each batch or individual image-based question to avoid memory bias. Prior to evaluation, models were uniformly prompted: “Hi [OpenAI/Grok/DeepSeek], you are a multimodal machine learning program. You are instructed to answer the following questions from either EyeQuiz or the StatPearls Ophthalmology question bank. I will copy each question stem, some of which may have images, with four to six possible answer choices. Please select the answer choice(s) that you believe is/are the correct answer to the best of your abilities and knowledge.”

### Data sources and question formats

The ophthalmology board-style questions for this study were sourced evenly from two commonly used platforms: *EyeQuiz* (*N* = 243) and *StatPearls* (*N* = 250). *EyeQuiz* is a freely available, open-access resource found online. Permission to use *StatPearls* content was explicitly granted. The dataset included True or False questions, single-answer multiple-choice questions, and multiple-answer multiple-choice questions. They were classified as image-containing (*N* = 74) and image-free (*N* = 419).

### Categorization approach

Each question was assigned to one of nine ophthalmology domains: (1) Lens and Cataract, (2) Cornea, External Disease, and Anterior Segment, (3) Glaucoma, (4) Neuro-Ophthalmology, (5) Oculofacial, Plastics, and Orbit, (6) Pediatric Ophthalmology and Strabismus, (7) Refractive Management and Surgery, (8) Retina, Vitreous, and Intraocular Tumors, and (9) Uveitis. Additionally, each question was classified based on cognitive complexity following Buckwalter’s schema using a three-level taxonomy: Level 1 (recognition and recall), Level 2 (comprehension and interpretation), and Level 3 (application of knowledge and problem-solving)^26,27^. Questions were also categorized by whether they contained images.

### Outcome measures

The primary outcome for this study was the overall accuracy percentage of correct responses from each AI model. Secondary outcomes specifically evaluated accuracy differences between questions with and without images, performance variability based on cognitive complexity, and comparisons across ophthalmology domains.

### Statistical techniques and data handling

McNemar’s test was utilized to compare model performances. Chi-square test was used to compare performance differences between question banks by the same model. Statistical significance was established at a P-value threshold of less than 0.05. Data recording and statistical analyses were performed using Microsoft Excel (Microsoft Corporation, Redmond, WA).

## Electronic supplementary material

Below is the link to the electronic supplementary material.

Supplementary Material 1

## Data Availability

The datasets generated during and/or analyzed during the current study are available from the corresponding author on reasonable request.
