# Supplementary material for: A comparative analysis of DeepSeek R1, DeepSeek-R1-Lite, OpenAi o1 Pro, and Grok 3 performance on ophthalmology board-style questions
Source: Sci Rep. 2025 Jul 2;15:23101. doi: 10.1038/s41598-025-08601-2 (PMC12215666; doi:10.1038/s41598-025-08601-2)
Supplement: Supplementary file 1 — Supplementary Material 1 [file 41598_2025_8601_MOESM1_ESM.pdf]

Supplementary Materials

Supplementary Figure S1

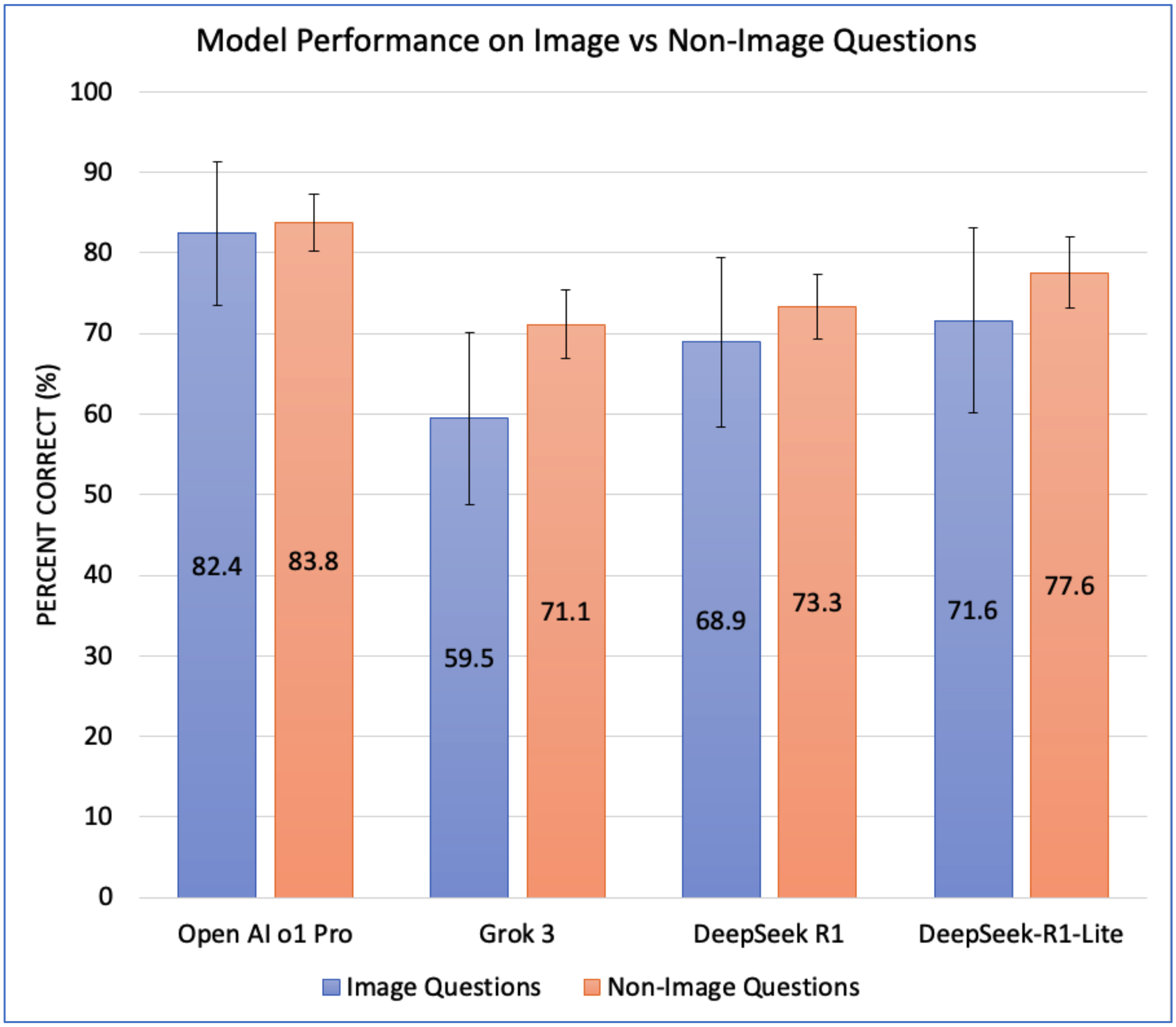

Supplementary Figure S2

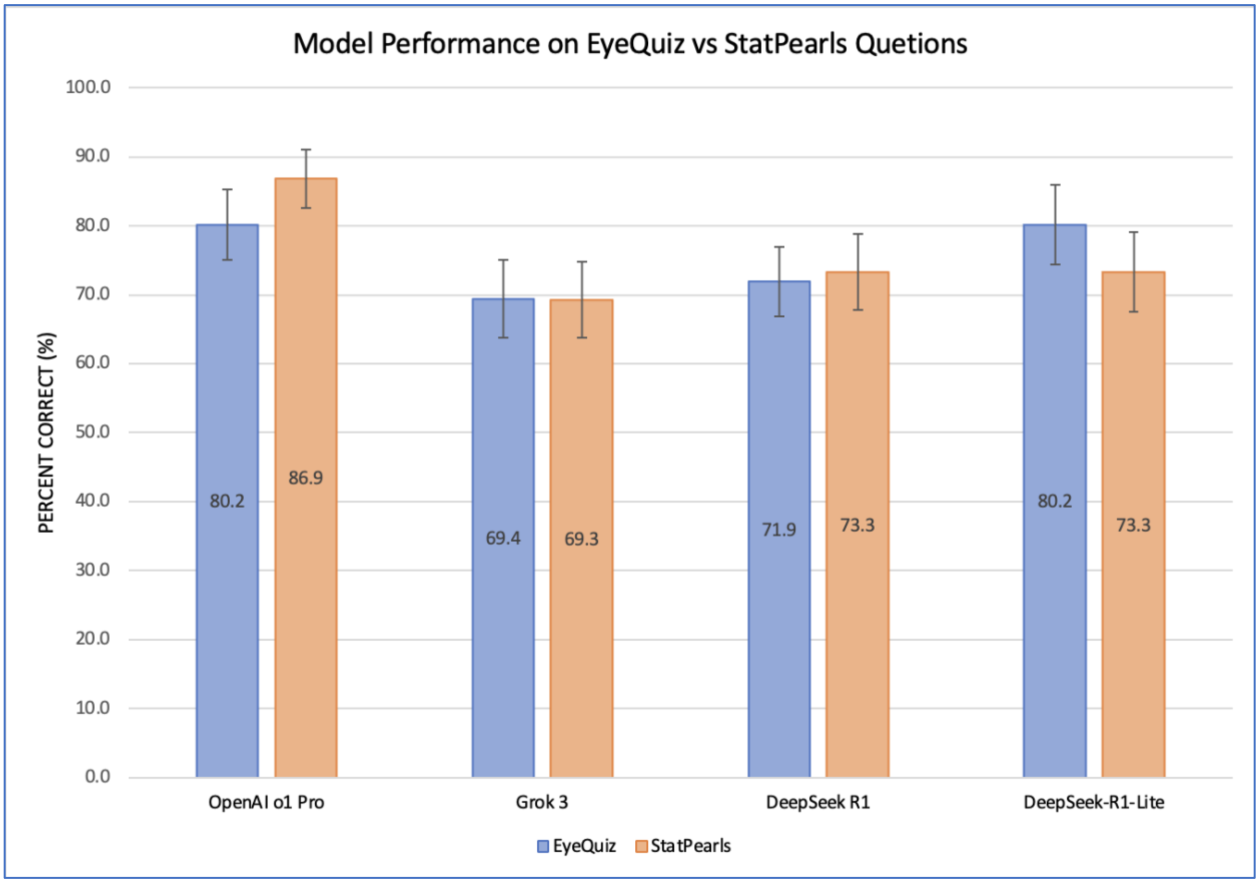

Supplementary Figure S3

Questions	Correct Answer (from *EyeQuiz* or *StatPearls*)	Image (Include if image-based question)	Question Category	Taxonomy Category (1=First Order 2=Second 3=Third)	GPT-o1 Pro Answer	Grok 3 Answer	DeekSeek Lite Answer	DeepSeek R1 Answer	GPT-o1 pro, Correct?	DeekSeek R1, correct?	DeepSeek Lite, correct?	Grok 3, correct?
What is the approximate risk of retinal detachment overall in the 5 year period after a cataract surgery?<br>A. 1%<br>B. 2%<br>C. 5%<br>D. 10%	A		8	1	A	B	A	A	Match	Match	Match	Not Match
A 60 year old patient presents with complaints of decreased vision. On exam, you find severe anterior basement membrane dystrophy and cataract. Which of the following tests would be most helpful in determining the main cause of the vision change?<br>A. Contact lens overrefraction<br>B. Corneal topography<br>C. Glare testing<br>D. Potential acuity meter	A		1	3	A	D	C	D	Match	Not Match	Not Match	Not Match
Indications for surgical excision of a pterygium include which of the following?<br>A. Persistent irritation<br>B. Irregular astigmatism<br>C. Difficulty with contact lens wear<br>D. Limitation of gaze<br>E. Cosmesis	A, B, C, D, E		2	2	A, B, C, D, E	A, B, C, D, E	A, B, C, D, E	A, B, D, E	Match	Not Match	Match	Match

Figure legends

Supplementary Figure S1. Performance of OpenAI o1, Grok 3, DeepSeek R1, and DeepSeek-R1-Lite on *StatPearls* and *EyeQuiz* questions stratified by questions containing images and questions not containing images. 95% confidence intervals are included.

Supplementary Figure S2. Performance of OpenAI o1, Grok 3, DeepSeek R1, and DeepSeek-R1-Lite, stratified by *StatPearls* and *EyeQuiz* Question banks.

Supplementary Figure S3. Example question-and-answer pairs including responses from each model from *EyeQuiz* questions.
